# Law enforcement personnel are willing to change, but report influencing beliefs and barriers to optimised dietary intake

**DOI:** 10.1186/s12889-020-09716-z

**Published:** 2020-11-02

**Authors:** Kristen MacKenzie-Shalders, Charlene Matthews, Joe Dulla, Robin Orr

**Affiliations:** 1 Nutrition and Dietetics, Faculty of Health Sciences and Medicine, Bond University, Robina, Australia; 2 Recruit Training Unit, Los Angeles County Sheriff’s Department, California, LA USA; 3 Tactical Research Unit, Bond University, Robina, Australia

**Keywords:** Police, Food choice, Behaviour, Nutrition, Health

## Abstract

**Bacmround:**

Law enforcement personnel have been recognized as having a high risk for several lifestyle-related health conditions which, in combination with the nature of their work (sedentary roles interspersed with intermittent high-intensity activity, shift work, and a high stress-load), can have a negative impact on their health. The aim of this study was to investigate the dietary habits and factors or barriers influencing these habits within a cohort of law enforcement personnel in the United States of America.

**Method:**

Cross-sectional data were obtained via validated paper-based surveys being the Perceived Barriers to Healthy Eating, Food Choice Questionnaire and Rapid Eating Assessment for Participants, Short Version.

**Results:**

A total of 159 participants (median age = 27 [range 19–60] years; 74% males) participated. Barriers to healthy eating included being busy and irregular working hours. Overall, 91% (*n* = 143) placed high importance on consuming nutritious food and 80% (*n* = 126) on food high in vitamins and minerals. A further 80% (*n* = 127) emphasized high protein content and 41% (*n* = 62) followed a high protein diet. Barriers to healthy eating included busy lifestyle (60%, *n* = 94), and irregular working hours (41%, *n* = 64). Overall, 80% (*n* = 127) were very willing to make changes in eating habits to be healthier.

**Conclusion:**

Law enforcement officers know what they should eat and report convenience and health the most important factors guiding their food choices. Knowing this, officers find challenges putting good dietary practices into practice due to factors like a busy lifestyle and irregular work hours. Reportedly “very willing” to make changes in their eating habits to be healthier, future interventions should focus on how to effect changes to their eating habits as opposed to focussing on what to eat.

## Background

Law enforcement personnel have been recognized as being prone to obesity [1] and having a high risk for several lifestyle-related health conditions [2–4]. The nature of their work, including sedentary roles interspersed with intermittent high-intensity activity, shift work, and a high stress-load can have a negative impact on their health and performance [5–7]. These personnel should maintain a requisite level of physical fitness to optimize their concentration, performance and safety; and as such while a focus on nutrition strategies that promote good health are important, a broader focus that encompasses strategies that optimize nutrition for performance is also warranted. Despite this, little is known about law-enforcement personnel’s dietary intake.

In law enforcement personnel, several studies have captured the duties involved in law enforcement and the corresponding impact on health [5–7]. In addition, there have been several studies linking socio-cultural and occupational factors with body composition and/or physical fitness [8–10]. However, few studies have directly explored dietary intake for this population [11–13]. Briley et al. evaluated the efficacy of a nutrition education component of a wellness program (pre- and post-implementation data) however, the baseline diet was not detailed [11]. Mumford et al. surveyed officers in law enforcement agencies regarding physical activity patterns, job characteristics, substance use, critical incidents, job-related stress, personal health and healthcare usage [12] and used a two-item measure of fruit and vegetable consumption on a typical day. They reported ~ 17.2% of respondents ate over 5 serves of fruit and vegetables per day, with a higher intake positively correlated with self-reported health status [12]. To date – the most comprehensive dietary study in law-enforcement personnel was performed by Gibson et al. [13]. The researchers performed a large-scale cohort study of law enforcement personnel worldwide by analysing participant data extracted from the Airwave Health Monitoring Study [13]. The Dietary Approaches to Stop Hypertension (DASH) score was used to determine dietary quality and a 7-day food record for dietary intake. The study reported dietary intake data and identified that a poor DASH score was associated with other negative lifestyle behaviours (inactivity, smoking and TV viewing). Furthermore, long working hours and high job strain (in men) increased the odds of reporting a dietary intake associated with cardiometabolic risk. Finally, the SHIELD study assessed law-enforcement personnel fruit and vegetable consumption using the National Cancer Institute’s fruit and vegetable all-day screener with additional questions to assess frequency, amount of dietary fat and self-reported healthy eating [14]. Rather than reporting dietary intake per se, the study focussed on changes in dietary scores pre and post intervention. Similarly and more broadly, several studies have demonstrated the successful impact of health or nutrition promotion programs on health attitudes and behaviors for athletic teams, firefighters, and law enforcement personnel [14–17].

While full, accurate dietary assessments can be difficult in law enforcement personnel, gaining an understanding of dietary patterns and diet quality is useful and can inform nutrition interventions. Correspondingly, a greater understanding of the factors that influence dietary patterns and diet quality in law enforcement personnel is needed to further inform potential interventions [18]. In particular, the identification of barriers to the consumption of healthy foods must be identified in the design of effective behaviour change interventions [19–21]. Law enforcement personnel are known to have a range of challenges including shift work, overtime, and lack of structured breaks due to their nature of their work [5–7, 13, 22] hence understanding their perception of barriers is useful.

To the authors’ knowledge, no research has been undertaken exploring the factors that influence food choice, beliefs, and diet quality (as measured by REAPS-S) in law enforcement personnel. Broadly, this study will investigate the dietary habits and food choices of a cohort of law enforcement personnel including custody assistants, civilian jailers, sworn deputies and police officers, and reserve peace officers in the United States of America (USA). The specific aims of this study are 1) to explore self-reported barriers to optimised dietary intake; 2) to report participant perceptions of factors that influence food choice; and 3) perform a rapid assessment of diet quality and willingness to change in a cohort of law enforcement personnel. The surveys chosen are validated, brief surveys that are suitable for use by time-poor law enforcement personnel [23–25]. It is anticipated that this research will provide valuable information for nutrition, performance, and health promotion strategies that support behaviour change, as well as organizational and systematic changes and future research that support this high-risk population.

## Methods

This single site, cross-sectional survey study was conducted in 2019 and evaluated data of law enforcement personnel in a large, metropolitan facility in the USA Cross-sectional data were obtained via validated paper-based surveys being the Perceived Barriers to Healthy Eating, Food Choice Questionnaire (FCQ) and Rapid Eating Assessment for Participants, Short Version (REAP-S). The questionnaires have been previously published [23–25] and are designed to measure perceived barriers/difficulties in eating a healthy diet, participants attitudes and beliefs on the factors that influence their dietary intake and a rapid first-choice dietary assessment indicating diet quality and willingness to change [23–25].

The Perceived Barriers to Healthy Eating questionnaire [24] was developed by a group of experts in attitudinal research and contains a list of 22 possible barriers for participants to select which they perceive as major difficulties in trying to eat a healthier diet. The Food Choice Questionnaire (FCQ) is a seminal tool which has been developed to measure multidimensional factors related to food choice at the individual level and through factor analysis health, mood, convenience, sensory appeal, natural content, price, weight control, familiarity and ethical concern emerged [23]. The questionnaire contains 36 questions and a five-point Likert Scale for responses ranging from ‘not important’ to ‘very important’. Since it was developed, the FCQ has been applied in a large number of countries from all continents [20], is used in a range of population groups [26–28]. The REAP-S was developed at the Albert Einstein College of Medicine of Yeshiva University and contains 13 questions regarding how often participants consume various foods or dietary patterns in an average week. There are three response categories of ‘usually/often’, ‘sometimes’, ‘rarely/never’ or ‘does not apply to me’ results indicating consumption frequency of various foods or dietary patterns. Survey scores provide an indicator of diet quality and the survey also asks how willing participants are to make changes in eating habits to be healthier; highlighting readiness to change.

Each of the surveys were provided to participants in a paper-based format to self-report following the obtainment of written informed consent. Ethics approval was provided by an International Review Board (IRB 15–074), and Bond University’s Human Research Ethics Committee (RO1927). The study has been reported according to STROBE guidelines [29] survey.

Participants were conveniently sampled; all law enforcement personnel present at a recruitment training unit over 20 weeks were invited to participate in the study. Three categories of personnel were captured: custody assistants or civilian jailers, sworn deputies and police officers and reserve peace officers. Inclusion criteria were a) adults aged ≥18 years, b) participants able to understand English, and c) participants provide voluntary consent. Participants were not required to declare medical conditions or health status but would have all passed a medical assessment to have been employed as law enforcement personnel.

Key demographic data were featured on the paper questionnaire, including self-reported height and weight which has been found to be accurate within a law enforcement population [30]. In addition, physical activity (resistance and endurance training frequency), and descriptive data on diets (diets followed, who prepares meals) were also collected. Height and weight were used to calculate body mass index via the following formula; weight (kg) / [height (m)]^2^ and classified as per the World Health Organization categories [31].

The validated paper-based survey responses were transferred to Statistical Package for the Social Science 25 (SPSS Statistics) where data were cleaned, categorized, and analysed. All continuous variables (age, weight, height) were not normally distributed hence are reported as median (range). Categorical variables were reported as frequency (n) and total percentage with all percentages referring to the valid data available for the variable. As a secondary analysis, a chi-square test was used for categorical variables to report associations between the different groups training; or Fisher’s exact test (two-sided) when results had ≥20% of cells with an expected count less than five. Significant difference and association were defined as *p* < 0.05 a priori. Survey results were synthesized and narratively discussed.

## Results

A total of 159 participants (median age = 27 [range 19–60] years; 74% males) undergoing training in the USA participated in the study. This included 40 custody assistants or civilian jailers, 99 sworn deputies and police officers and 20 reserve peace officers representing a cross-section of law enforcement personnel. All 159 participants answered all questionnaires. Demographic related characteristics of grouped participants are displayed in Table 1. Comparisons between group demographics are in Additional file 1. With the exception of a significant difference (*p* < 0.001) in the median age of reserve peace officers 42 (22–60) years compared to custody assistants and sworn deputies (median age 25 (19–39) and 26 (20–50) years respectively); there were no other significant demographic differences between groups. All law enforcement personnel reported doing regular resistance and aerobic exercise with the majority doing ≥3 sessions per week (*n* = 108, 69% and *n* = 117, 73% respectively). There was a significant difference between groups as fewer reserve peace officers reported ≥3 sessions per week of resistance (*n* = 8, 40%) and aerobic (n = 10, 50%) exercise compared to custody assistants (*n* = 24, 62% and *n* = 25, 63% respectively) and sworn deputies (*n* = 76, 77% and *n* = 82, 82% respectively) (*p* = 0.036 for resistance and *p* = 0.004 aerobic respectively). Additionally, there were differences in who most often prepares meals with reserve peace officers taking responsibility less (*n* = 7, 35%) compared to custody assistants (*n* = 22, 57.9%) and sworn deputies (48, 51.1%) (*p* = 0.003).

**Table 1:** Demographic related characteristics and descriptive results of the 159 participants (*n*= available data of the 159 participants for that variable)

Characteristic	n (%)
**Gender** (***n*=156)**	
Male	116 (74.0%)
Female	40 (26.0%)
**Age (years) median (range)** (***n*=158)**^**a**^	27 (19–60)
**Weight (lbs) median (range)** (***n*=159)**^**a**^	175 (110–280)
**Weight (kg) median (range)** (***n*=159)**^**a**^	79 (50–127)
**Body Mass Index (kg/m**^**2**^**)** (***n*=159**)	
< 18.5	0 (0.0%)
18.5–24.9	52 (32.7%)
25.0–29.9	82 (51.5%)
> 30	25 (15.7%)
**Height (ft) median (range)** (***n*=159)**^**a**^	5.8 (4.6–6.5)
**Height (m) median (range)** (***n*=159)**^**a**^	1.8 (1.4–2.0)
**Resistance training** (***n*=158)**^**b**^	
1 session per week	15 (9.0%)
2 sessions per week	35 (22.0%)
3 sessions per week	58 (37.0%)
≥ 4 sessions per week	50 (32.0%)
**Endurance/aerobic** (***n*=159)**^**b**^	
1 session per week	6 (4.0%)
2 sessions per week	36 (23.0%)
3 sessions per week	64 (40.0%)
≥ 4 sessions per week	53 (33.0%)
**Follows a special diet** (***n*=153)**^**c**^	
No	71 (46.4%)
Yes	82 (53.6%)
**Diets followed** (***n*=153)** ^**d**^	
High protein	62 (40.5%)
Low-carb	22 (14.4%)
Salt-reduced	17 (11.1%)
No sugar	13 (8.5%)
High-carb	12 (7.8%)
Low-calorie	10 (6.5%)
Carbohydrate cycling	7 (4.6%)
Other^e^	23 (15.2%)
**Who most often prepares meals** (***n*=152)**	
Only me	77 (50.7%)
Family member	45 (29.6%)
Partner	22 (14.5%)
Other^e^	8 (5.3%)

^a^ All continuous variables (age, weight, height) were not normally distributed hence are reported as median (range). ^b^ Training session length not defined. ^c^ The reason for following a special diet could be a lifestyle choice or due to a medical condition i.e. diabetes. ^d^ Participants were to select all that applied hence non accumulative percentage. ^e^ Other includes: vegetarian, Atkins, gluten free, vegan, dairy free, paleo, lacto-ovo vegetarian, food allergy or intolerance, high calorie. ^f^ Other includes: special food service and restaurants

Results on the perceived barriers to healthy eating can be seen in Table 2. Main barriers included busy lifestyle (60%, *n* = 94), irregular working hours (41%, *n* = 64), lengthy food preparation (35%, *n* = 55), price of healthy food (32%, *n* = 51) and cooking skills (30%, *n* = 48). Additional file 1 features between-group differences.

**Table 2:** Perceived barriers to eating a healthier diet: The Perceived Barriers to Healthy Eating ranked responses

What barriers to healthy eating can you identify with? n (%)	Overall
Busy lifestyle	94 (59.5%)
Irregular working hours	64 (40.5%)
Lengthy preparation	55 (34.8%)
Price of healthy foods	51 (32.3%)
Cooking skills	48 (30.4%)
Not knowing enough about healthy eating	46 (29.1%)
Not enough food to satisfy hunger	31 (19.6%)
Limited choice when I eat out	30 (19.0%)
Giving up foods I like	25 (15.8%)
Willpower	24 (15.2%)
Unappealing food	20 (12.7%)
Taste preferences of family and friends	20 (12.7%)
Healthy food is more perishable	17 (10.8%)
Strange or unusual foods	15 (9.5%)
Experts keep changing their minds	10 (6.3%)
Storage facilities	9 (5.7%)
Limited cooking facilities	8 (5.1%)
Healthy options not available canteen/home	7 (4.4%)
I don’t want to change my eating habits	2 (1.3%)
Healthy food more awkward to carry from shops	2 (1.3%)
Too great a change from my current diet	1 (0.6%)
Feeling conspicuous amongst others	0 (0.0%)

Results from The FCQ can be seen in Table 3. The majority of participants placed high importance on consuming nutritious food that keeps them heathy and is high in vitamins and minerals (91%, *n* = 143 and 80%, *n* = 126 respectively), as well as having a high protein content (80%, *n* = 127) (Table 3). Nearly half of participants followed a high protein diet (41%, *n* = 62). (Table 1). Of those that report following a high-protein diet only 4 participants reported usually/often eating more than 8 oz of meat a day (6.5%, *n* = 4). Overall, convenience and health were the most important factors influencing food choice (Table 3).

**Table 3:** Factors related to food choice at the individual level; The Food Choice Questionnaire (FCQ) responses (*n*= available data of the 159 participants for that variable)

FCQ factors n (%)	Not Important	Low Importance	Neutral	Important	Very Important
Keeps me healthy (*n*=159)^a^	0 (0.0%)	2 (1.3%)	12 (7.5%)	62 (39.0%)	83 (52.2%)
Nutritious (*n*=158)^a^	1 (0.6%)	1 (0.6%)	13 (8.2%)	63 (39.9%)	80 (50.6%)
Easily available (*n*=159)^a^	1 (0.6%)	4 (2.5%)	23 (14.5%)	66 (41.5%)	65 (40.9%)
Keeps me awake and alert (*n*=159)^a^	4 (2.5%)	7 (4.4%)	21 (13.2%)	68 (42.8%)	59 (37.1%)
High in vitamins/minerals (*n*=159)^a^	1 (0.6%)	5 (3.1%)	27 (17.0%)	68 (42.8%)	58 (36.5%)
Tastes good (*n*=159)^a^	2 (1.3%)	5 (3.1%)	18 (11.3%)	77 (48.4%)	57 (35.8%)
Good value for money (*n*=159)^a^	1 (0.6%)	6 (3.8%)	29 (18.2%)	66 (41.5%)	57 (35.8%)
Helps control my weight (*n*=159)^a^	6 (3.8%)	12 (7.5%)	37 (23.3%)	47 (29.6%)	57 (35.8%)
High in protein (*n*=159)^a^	0 (0.0%)	6 (3.8%)	26 (16.4%)	73 (45.9%)	54 (34.0%)
Easy to prepare (*n*=159)^a^	4 (2.5%)	8 (4.0%)	41 (25.8%)	54 (34.0%)	52 (32.7%)
Can be bought close to where I live or work (*n*=159)^a^	3 (1.9%)	13 (8.2%)	29 (18.2%)	66 (41.5%)	48 (30.2%)
Is good for my skin/teeth/hair/nails (*n*=159)^a^	2 (1.3%)	7 (4.4.%)	40 (25.2%)	63 (39.6%)	47 (29.6%)
Contains natural ingredients (*n*=158)^a^	2 (1.3%)	6 (3.8%)	32 (20.3%)	72 (45.6%)	46 (29.1%)
Can be cooked very simply (*n*=159)^a^	4 (2.5%)	12 (7.5%)	29 (18.2%)	73 (45.9%)	41 (25.8%)
Makes me feel good (*n*=159)^a^	7 (4.4%)	15 (9.4%)	39 (24.5%)	60 (37.7%)	38 (23.9%)
Not expensive (*n*=159)^a^	5 (3.1%)	11 (6.9%)	55 (34.6%)	52 (32.7%)	36 (22.6%)
No additives (*n*=157)^a^	3 (1.9%)	18 (11.5%)	62 (39.5%)	45 (28.7%)	29 (28.7%)
No artificial ingredients (*n*=159)	2 (1.3%)	21 (13.2%)	65 (40.9%)	44 (27.7%)	27 (17.0%)
Low in fat (*n*=159)	5 (3.1%)	25 (15.7%)	59 (37.1%)	47 (29.6%)	23 (14.5%)
Cheap (*n*=159)	7 (4.4%)	16 (10.1%)	65 (40.9%)	48 (30.2%)	23 (14.5%)
Takes no time to prepare (*n*=158)	5 (3.2%)	21 (13.3%)	60 (38.0%)	49 (31.0%)	23 (14.6%)
Helps me relax (*n*=159)	17 (10.7%)	29 (18.2%)	61 (38.4%)	30 (18.9%)	22 (13.8%)
Helps me cope with stress (*n*=158)	29 (18.4%)	28 (17.7%)	57 (36.1%)	24 (15.2%)	20 (12.7%)
Has a pleasant texture (*n*=159)	6 (3.8%)	22 (13.8%)	59 (37.1%)	53 (33.3%)	19 (11.9%)
Low in calories (*n*=159)	7 (4.4%)	27 (17.0%)	73 (45.9%)	35 (22.0%)	17 (10.7%)
High in fibre (*n*=159)	5 (3.1%)	19 (11.9%)	70 (44.0%)	49 (30.8%)	16 (10.1%)
Familiar to me (*n*=159)	13 (8.2%)	28 (17.6%)	44 (27.7%)	61 (38.4%)	13 (8.2%)
Smells nice (*n*=159)	9 (5.7%)	27 (17.0%)	50 (31.4%)	60 (37.7%)	13 (8.2%)
Helps me cope with life (*n*=159)	30 (18.9%)	30 (18.9%)	58 (36.5%)	28 (17.6%)	13 (8.2%)
Is what I usually eat (*n*=159)	20 (12.6%)	27 (17.0%)	61 (38.4%)	39 (24.5%)	12 (7.5%)
Environmentally friendly packaging (*n*=158)	20 (12.7%)	37 (23.4%)	55 (34.8%)	34 (21.5%)	12 (7.6%)
Has the country of origin clearly marked (*n*=159)	57 (35.8%)	35 (22.0%)	45 (28.3%)	14 (8.8%)	8 (5.0%)
Comes from countries I approve of politically (*n*=159)	70 (44.0%)	33 (20.8%)	41 (25.8%)	9 (5.7%)	6 (3.8%)
Looks nice (*n*=159)	33 (20.8%)	36 (22.6%)	54 (34.0%)	30 (18.9%)	6 (3.8%)
Is like the food I ate when I was a child (*n*=159)	50 (31.4%)	46 (28.9%)	50 (31.4%)	8 (5.0%)	5 (3.1%)
Cheers me up (*n*=159)	21 (13.2%)	17 (10.7%)	59 (37.1%)	39 (24.5%)	23 (14.5%)

^a^ Indicates the FCQ factor has an accumulative percentage ≥ 50% for ‘important’ and ‘very important’

Results from the REAP-S survey including intake of core food groups, discretionary foods, and dietary patterns can be seen in Table 4. Scores from the REAP-S varied with 20% scoring between 19 and 27 (*n* = 32), 70% scoring 28–34 (*n* = 112) and 10% scoring 35–37 (*n* = 15). When asked ‘how willing are you to make changes in your eating habits in order to be healthier?’ the majority reported being very willing (80%, *n* = 127) followed by 14% willing (*n* = 22), 4% neutral (*n* = 7), 1% not very willing (n = 1) and 1% not willing at all (n = 2).

**Table 4:** Consumption frequency or dietary patterns: Rapid Eating Assessment for Participants Short Version (REAP-S) responses (*n*= available data of the 159 participants for that variable)

In an average week, how often do you? n (%)	Usually/Often	Sometimes	Rarely/Never	Does not apply to me ^a^
1. Skip breakfast? (*n*=157)	38 (24.2%)	53 (33.8%)	66 (42.0%)	
2. Eat 4 or more meals from sit-down or take out restaurants? (*n*=158)	10 (6.3%)	73 (46.2%)	75 (47.5%)	
3. Eat less than 2 servings of whole grain products or high fibre starches a day? (*n*=157)	18 (11.5%)	66 (42.0%)	73 (46.5%)	
4. Eat less than 2 servings of fruit a day? (*n*=159)	25 (15.7%)	80 (50.3%)	54 (34.0%)	
5. Eat less than 2 servings of vegetables a day? (*n*=155)	12 (7.7%)	88 (56.8%)	55 (35.5%)	
6. Eat or drink less than 2 servings of milk, yogurt, or cheese a day? (*n*=157)	44 (28.0%)	71 (45.2%)	42 (26.8%)	
7. Eat more than 8 oz of meat, chicken, turkey or fish per day? (*n*=159)	76 (47.8%)	56 (35.2%)	23 (14.5%)	4 (2.5%)
8. Use regular processed meats? (*n*=158)	11 (7.0%)	60 (38.0%)	74 (46.8%)	13 (8.2%)
9. Eat fried foods such as fried chicken, fried fish, French fries, fried plantains …? (*n*=157)	10 (6.4%)	77 (49.0%)	70 (44.6%)	
10. Eat regular potato chips, nacho chips, corn chips, crackers, regular popcorn, nuts instead of pretzels, low-fat chips or low- fat crackers, air-popped popcorn? (*n*=158)	15 (9.5%)	56 (35.4%)	71 (44.9%)	16 (10.1%)
11. Add butter, margarine or oil to bread, potatoes, rice or vegetables at the table? (*n*=158)	24 (15.2%)	36 (22.8%)	98 (62.0%)	
12. Eat sweets like cake, cookies, pastries, donuts, muffins, chocolate and candies more than 2 times per day? (*n*=157)	3 (1.9%)	40 (25.5%)	114 (72.6%)	
13. Drink 16 oz or more of non-diet soda, fruit drink/punch or Kool-Aid a day? (*n*=156)	4 (2.6%)	20 (12.8%)	132 (84.6%)	

^a^ ‘Does not apply to me’ was an option for questions 7–8 and 10. Responding with this indicates participants ‘rarely eat meat, chicken, turkey or fish’; ‘rarely eat processed meats’; and/or ‘rarely eats these snack foods’ respectively. Where participants responded with ‘does not apply to me’ on a question that did not offer this as a response option, data has been considered as missing data (*n* = 10)

## Discussion

Several studies have demonstrated the successful impact of health and nutrition promotion programs on health attitudes and behaviours for athletic teams, firefighters, and law enforcement personnel [14–17]. However, to effectively target nutrition interventions for law enforcement personnel, it is important that they are tailored specifically for the requirements of this high-risk population. This study provides insight into the factors that influence food choice, perceived barriers to healthy eating, dietary patterns, and willingness to change in law enforcement personnel undergoing training.

Firstly, and importantly, the majority of law enforcement personnel in this study reported being “very willing” to make changes in their eating habits to be healthier (80%, *n* = 127). However, qualitative studies within law enforcement officers found they believed their attempts to make healthier dietary changes were often sabotaged by the “nature of the job” including irregular work hours and unpredictable events [32]. Therefore, well-intentioned nutrition programs that instruct personnel on *what* they should eat as a base without a practical and tailored approach may not be successful. Instead, focusing on *how* to change is beneficial, and is part of the premise of beneficial programs such as the SHIELD program in law enforcement officers [14]. The team-based health promotion program was found to be feasible and effective at 6 months in improving diet across a range of variables (e.g. fruit and vegetable consumption and healthy eating) and in improving other health-related variables (e.g. sleep quality and quantity and stress levels). Overall, the program was feasible, effective, and durable for improving dietary changes.

While education is an important strategy for optimizing dietary intake, few law enforcement personnel identified not knowing enough about healthy eating as a barrier (*n* = 46, 29%). While the focus of the study was not on dietary intake per se; diet quality was indicated from the REAP-S. Twenty percent scored between 19 and 27 (*n* = 32) indicating lower diet quality, 70% scored from 28 to 34 (*n* = 112) and 10% scored 35–37 (*n* = 15) indicating a higher diet quality. Interestingly, it has been shown that perceived diet quality in American adults is associated with actual diet quality [33]. These findings suggest that while law enforcement personnel may have an awareness of what they should be eating, they may not always put this knowledge into practice. For example, the majority of participants placed a high importance on consuming nutritious food that kept them heathy and was high in vitamins and minerals (91%, *n* = 143 and 80%, *n* = 126 respectively), however their fruit and vegetable intake were low with more than half reporting ‘usually’ or ‘sometimes’ eating less than two serves a day (*n* = 105, 66% and *n* = 100, 65% respectively). This intention-behaviour gap may highlight that focusing on factors beyond nutrition knowledge that enable behaviour change (e.g. time management and organization, quick-healthy foods) may be important.

Collectively factors of convenience were reported as the most influential over food choice, and barriers to healthy eating included being busy and irregular working hours (*n* = 64, 41% and *n* = 94, 60% respectively). These food choice behaviours were expressed in the personnel’s dietary patterns; for example, 57% of respondents skipped breakfast often or sometimes (*n* = 91) and similarly, over half of the participants reported often or sometimes eating four or more meals from sit-down or take out restaurants (52%, *n* = 83). Law enforcement personnel are subject to shift work, overtime and often do not have structured breaks due to their nature of their work. As a result, law enforcement personnel may not adhere to conventional mealtimes. In addition, they may have an increased reliance on snacking and take-away food which include proportionally higher quantities of sweet and savory bakery products, soft drinks, juices and other non-alcoholic beverages compared to eating at home [34]. Interestingly, of the individuals who reported that they were responsible for their meal preparation (*n* = 77, 48.4%), only 5% reported eating out often compared to 46% who reported that when their partner cooked and 56% when a family member cooked. This reinforces a benefit for law enforcement personnel to be actively involved in their own food preparation, which may translate to better food preparation knowledge and organization.

While food choices reported by law enforcement personnel are relatively in line with nutritional recommendations within the USA, targeted support for this population is warranted. Considering that this population is at increased risk of several lifestyle-related health conditions, has increased physical requirements due to the demanding physical profession, and requires concentration in high-stress situations, optimizing dietary intake is of high importance as is maintain physical fitness. Law enforcement is a demanding profession and can place physical stress on personnel as they may be required to push, pull, lift, carry, or drag objects or people at any time during their shift [22]. Furthermore, daily tasks are often performed whilst carrying and wearing an external load that can weight around 10 kg [35]. In order to perform these physical tasks effectively, especially while wearing this external load, police officers are required to have a requisite amount of physical fitness [36, 37]. Considering this, while all law enforcement personnel within this study reported doing regular resistance exercise and aerobic exercise with the majority doing ≥3 sessions per week (*n* = 108, 69% and *n* = 117, 73% respectively), several dietary behaviours would not be considered optimal from a performance perspective. For example, a proportion of personnel rarely consumed more than 8 oz of protein-containing foods per day (*n* = 23, 15%) and the majority usually or sometimes consumed less than two serves of dairy-contain foods per day (*n* = 115, 73%); despite the majority believing protein intake was important or very important. When asked if they followed a special diet, 14% of participants reported following a low-carb diet (*n* = 22), 9% a no sugar diet (*n* = 13) and 7% a low-calorie diet (*n* = 12); indicating a tendency for some participants to eliminate food groups. Suboptimal dietary energy intake, and corresponding decreases in macro and micronutrients, are likely to influence performance in law enforcement personnel undergoing training [38, 39]. Furthermore, conscious restriction is often employed to control body weight however, imbalances in macronutrients may stimulate regulatory signals in the body, leading to overeating [40–42]. Where this dietary restriction is excessive or is coupled with concerning dietary beliefs and behaviours, an individualized approach with a dietitian or health professional is warranted.

There are several published team-based health and nutrition promotion programs supporting this population or similar population groups. Some reported topics included how to replace unhealthy fast food with healthy alternatives i.e. “a fast food makeover”, how to shop and cook healthy on a budget, how to reduce calories in snacks, lunches that can be brought from home “brownbag makeover” and counselling [14, 17, 43]. Additional strategies that have been reported have included time management or motivational tools including reminders, self-monitoring and reinforcement [43]. These types of strategies are supported by the current study. In addition, at a systematic or organizational level, further strategies for this workforce may include vehicle design and support for food and drink, choice architecture, healthy on-site food service provision and agreements/discounts with local healthy cafes and restaurants.

### Strengths and limitations

The main strength of this study is the comprehensive and rigorous data collection via different validated surveys providing a broader description of diet pattern, diet quality, beliefs and motives as well as current barriers to healthy eating within a cohort of law enforcement personnel in the USA. With greater insight into these habits, tailored interventions at an individual and systematic/organizational level can be designed thereby increasing the chance of effectively initiating, and potential maintaining, enhanced nutrition behaviours. Noting this strength, several limitations require consideration. The current study was a single site study of law enforcement personnel undergoing training which can impact on its generalizability, for example its findings may not be applicable to law enforcement personnel in rural areas, in specialist roles, or after longer periods of service. To mitigate this limitation, the study does provide key information to inform future studies or interventions. A convenience sample was required due to difficulties capturing data from this population and the lack of current research in the area. While the study was undertaken in a relatively small sample size which did not support between-group analyses; it was undertaken in a group that are not commonly studied due to access and time constraints and featured a high response rate. While care was taken to choose appropriate tools, the surveys were not directly validated for this population. Though the validated tools are designed for self-reporting, some components may have been inaccurate or subject to biases. For example, BMI was included to provide demographic information on the personnel, but its limitations for an active population are acknowledged. To the authors’ knowledge, the validated studies are the best available for the population being studied at the time of data collection.

## Conclusion

In law enforcement, enhancing officer physical performance, health, and safety, as well as injury mitigation and management, is important and suboptimal nutrition can directly threaten these factors. This study aimed to investigate the readiness to change, dietary habits, factors that influence food choice, and barriers to healthy eating in a cohort of USA law enforcement personnel undergoing training. This research provides descriptions of the habits, barriers and factors influencing healthy eating of law enforcement personnel and highlighted several dietary and behavioural areas of concern that are specific to this high-risk group. Practically, the study has supported that; 1) Nutrition and health programs should recognize and support law enforcement personnel’s motivation and readiness to change. 2) Nutrition and health programs can include content to enhance knowledge; but this should be supported by practical strategies that support behaviour change and address reported barriers to health eating i.e. focusing on *how* to change is beneficial. 3) While individual responsibility is important, systemic, and organizational approaches are important as some barriers to health eating (e.g. shift work, access to good food issues) are outside of the law enforcement personnel’s control.

Further qualitative and quantitative research would be beneficial to further support the development of targeted nutrition programs and strategies for law enforcement personnel. For example, increased periods of service or decreasing physical activity may impact readiness to change, food behaviours, factors that influence food choice, and barriers to healthy eating for law enforcement personnel. In addition, while not an exhaustive list, exploring differences in gender, age, rural versus metropolitan, roles/ranks, or responses in periods of heightened stress may be useful. This research has provided valuable information for nutrition, performance, and health promotion strategies that support behaviour change, as well as organizational and systematic changes that support this high-risk population.

## Supplementary information

**Additional file 1.** Inter-group comparison of demographic related and descriptive results.

## Data Availability

The datasets used and/or analysed during the current study are available from the corresponding author on reasonable request.

## Abbreviations

USA: United States of America
FCQ: Food Choice Questionnaire
REAP-S: Rapid Eating Assessment for Participants – shortened version
IRB: International Review Board
STROBE: Strengthening the Reporting of Observational Studies in Epidemiology
SPSS: Statistical Package for the Social Science
SHIELD: Safety & Health Improvement: Enhancing Law Enforcement Departments
