# Supplementary material for: Law enforcement personnel are willing to change, but report influencing beliefs and barriers to optimised dietary intake
Source: BMC Public Health. 2020 Nov 2;20:1638. doi: 10.1186/s12889-020-09716-z (PMC7607818; doi:10.1186/s12889-020-09716-z)
Supplement: Supplementary file 1 — Additional file 1. Inter-group comparison of demographic related and descriptive results. [file 12889_2020_9716_MOESM1_ESM.docx]

**Additional file 1.** Inter-group comparison of demographic related and descriptive results

| **Characteristic n (%)** | **Overall (N=159)** | **Custody Assistants and Civilian Jailers (n=40)** | **Sworn Deputies and Police Officers**  **(n=99)** | **Reserve Peace Officers (n=20)** | **p-value** |
| --- | --- | --- | --- | --- | --- |
| Gender |  |  |  |  |  |
| Male | 116 (74.0%) | 30 (75.0%) | 70 (71.0%) | 16 (88.9%) | 0.291 |
| Female | 40 (26.0%) | 10 (25.0%) | 28 (29.0%) | 2 (11.1%) |  |
| Age (years) median (range) | 27 (19-60) | 25 (19-39) | 26 (20-50) | 42 (22-60) | <0.001* |
| Weight (lbs) median (range) | 175 (110-280) | 177 (130-270) | 170 (110-235) | 193 (130-280) | 0.450 |
| Body Mass Index (kg/m^2^) | |  |  |  |  |
| <18.5 | 0 (0%) | 0 (0.0%) | 0 (0.0%) | 0 (0.0%) | 0.526 |
| 18.5–24.9 | 52 (32.7%) | 11 (27.5%) | 36 (36.4%) | 5 (25.0%) |  |
| 25.0–29.9 | 82 (51.5%) | 24 (60.0%) | 48 (48.5%) | 10 (50.0%) |  |
| >30 | 25 (15.7%) | 5 (12.5%) | 15 (15.2%) | 5 (25.0%) |  |
| Height (ft) median (range) | 5.8 (4.6-6.5) | 5.7 (5.0-6.3) | 5.7(4.6-6.5) | 5.8 (5.3-6.3) | 0.883 |
| Resistance training | |  |  |  |  |
| 1 session/week | 15 (9.0%) | 4 (10.0%) | 7 (7.1%) | 4 (20.0%) | 0.036* |
| 2 sessions/week | 35 (22.0%) | 11 (28.0%) | 16 (16.2%) | 8 (40.0%) |  |
| 3 sessions/week | 58 (37.0%) | 16 (41.0%) | 37 (37.4%) | 5 (25.0%) |  |
| ≥4 sessions/week | 50 (32.0%) | 8 (21.0%) | 39 (39.4%) | 3 (15.0%) |  |
| Endurance/aerobic |  |  |  |  |  |
| 1 session/week | 6 (4.0%) | 0 (0.0%) | 4 (4.0%) | 2 (10.0%) | 0.004* |
| 2 sessions/week | 36 (23.0%) | 15 (37.5%) | 13 (13.1%) | 8 (40.0%) |  |
| 3 sessions/week | 64 (40.0%) | 16 (40.0%) | 43 (43.4%) | 5 (25.0%) |  |
| ≥4 sessions/week | 53 (33.0%) | 9 (22.5%) | 39 (39.4%) | 5 (25.0%) |  |
| Follows a special diet | |  |  |  |  |
| No | 71 (46.4%) | 16 (41.0%) | 44 (46.8%) | 11 (55.0%) | 0.590 |
| Yes | 82 (53.6%) | 23 (59.0%) | 50 (53.2%) | 9 (45.0%) |  |
| Diets followed^a^ | | |  |  |  |
| High protein | 62 (40.5%) | 17 (43.6%) | 39 (41.5%) | 6 (30.0%) | 0.575 |
| Low carbohydrate | 22 (14.4%) | 6 (12.8%) | 14 (14.9%) | 3 (15.0%) | 0.950 |
| Salt reduced | 17 (11.1%) | 6 (15.4%) | 10 (10.6%) | 1 (5.0%) | 0.520 |
| No sugar | 13 (8.5%) | 5 (12.8%) | 6 (6.4%) | 2 (10.0%) | 0.443 |
| High carbohydrate | 12 (7.8%) | 3 (7.7%) | 9 (9.6%) | 0 (0.0%) | 0.551 |
| Low calorie | 10 (6.5%) | 5 (12.8%) | 4 (4.3%) | 1 (5.0%) | 0.179 |
| Carbohydrate cycling | 7 (4.6%) | 0 (0.0%) | 7 (7.4%) | 0 (0.0%) | 0.138 |
| Other^b^ | 23 (15.2%) | 3 (7.7%) | 17 (18.2%) | 3 (15.0%) | 0.009* |
| Who most often prepares meals | |  |  |  |  |
| Only me | 77 (50.7%) | 22 (57.9%) | 48 (51.1%) | 7 (35.0%) | 0.003* |
| Family member | 45 (29.6%) | 6 (15.8%) | 35 (37.2%) | 4 (20.0%) |  |
| Partner | 22 (14.5%) | 8 (21.1%) | 9 (9.6%) | 5 (25.0%) |  |
| Other^c^ | 8 (5.3%) | 2 (5.3%) | 2 (2.1%) | 4 (21.0%) |  |

^a^ Participants were asked to select all diets that applied hence non accumulative percentage

^b^ Other includes: vegetarian, Atkins, gluten free, vegan, dairy free, paleo, lacto-ovo vegetarian, food allergy/intolerance, high calorie

^c^ Other includes: special food service and restaurants

* Significant difference set at p-value ≤0.05 analyzed via a chi-square test for categorical variables or Fisher’s exact test (two-sided) when results had ≥20% of cells with an expected count less than five.
